# Simulated Birdwatchers’ Playback Affects the Behavior of Two Tropical Birds

**DOI:** 10.1371/journal.pone.0077902

**Published:** 2013-10-11

**Authors:** J. Berton C. Harris, David G. Haskell

**Affiliations:** 1 Fundación de Conservación Jocotoco, Quito, Ecuador; 2 Woodrow Wilson School of Public and International Affairs, Princeton University, Princeton, New Jersey, United States of America; 3 Department of Biology, the University of the South, Sewanee, Tennessee, United States of America; Smithsonian's National Zoological Park, United States of America

## Abstract

Although recreational birdwatchers may benefit conservation by generating interest in birds, they may also have negative effects. One such potentially negative impact is the widespread use of recorded vocalizations, or “playback,” to attract birds of interest, including range-restricted and threatened species. Although playback has been widely used to test hypotheses about the evolution of behavior, no peer-reviewed study has examined the impacts of playback in a birdwatching context on avian behavior. We studied the effects of simulated birdwatchers’ playback on the vocal behavior of Plain-tailed Wrens *Thryothorus euophrys* and Rufous Antpittas *Grallaria rufula* in Ecuador. Study species’ vocal behavior was monitored for an hour after playing either a single bout of five minutes of song or a control treatment of background noise. We also studied the effects of daily five minute playback on five groups of wrens over 20 days. In single bout experiments, antpittas made more vocalizations of all types, except for trills, after playback compared to controls. Wrens sang more duets after playback, but did not produce more contact calls. In repeated playback experiments, wren responses were strong at first, but hardly detectable by day 12. During the study, one study group built a nest, apparently unperturbed, near a playback site. The playback-induced habituation and changes in vocal behavior we observed suggest that scientists should consider birdwatching activity when selecting research sites so that results are not biased by birdwatchers’ playback. Increased vocalizations after playback could be interpreted as a negative effect of playback if birds expend energy, become stressed, or divert time from other activities. In contrast, the habituation we documented suggests that frequent, regular birdwatchers’ playback may have minor effects on wren behavior.

## Introduction

Recreational birdwatchers potentially benefit conservation by generating interest in birds and natural habitats. For example, citizen scientist birdwatchers are the primary data collectors for biodiversity monitoring programs such as the North American Breeding Bird Survey [1] and eBird (www.ebird.org), and birdwatchers collect point locality data that supplements dwindling museum collecting effort [2,3]. In addition, touring birdwatchers–the largest, best-educated, and wealthiest ecotourist group–support economic networks in the developing world, and promote land conservation as a result [4,5]. Although birdwatching likely has a net positive influence on birds and habitats, some birdwatchers’ actions may negatively affect the environment (e.g. [6]), yet birdwatchers’ impacts are rarely quantified. 

One potentially harmful activity of birdwatchers is the use of recorded vocalizations, or “playback,” to attract species of interest. Territorial songs or contact calls are used to either draw a species into the open or stimulate a bold territorial response [7]. Playback is used by recreational birdwatchers and tour companies worldwide, but it is especially common in the tropics where secretive, forest interior species such as the Grallaridae are difficult to locate without playback. Some tropical destinations such as the Canopy Tower, Panama, can daily receive dozens of birders and annually receive over ten tours from a single tour company [8]. Birdwatchers often select tours based on previous bird lists, and they expect to see the rare, and often threatened, birds located on the previous tour. In many cases, difficult species are located with playback which may be used for over 1 hour until every person in the tour group has seen the bird well (J.B.C.H. pers. obs.).

The possible consequences of simulated territorial intrusions produced by birdwatchers’ playback are poorly understood. Birds may take time away from foraging, expend energy, or make themselves vulnerable to predation or extra-pair copulations when responding to playback [9,10]. Playback (in a non-birdwatching context) may cause elevated corticosterone and testosterone levels in birds [11,12], which could have fitness consequences [13]. Furthermore, one study found that female Black-capped Chickadees *Poecile atricapilla* listen to interactions between males and broadcast vocalizations, and tend not to mate with high-ranking males that lose standoffs with simulated aggressive males [14].

In contrast to the above cases, birdwatchers’ playback could have negligible or even positive impacts on birds. For example, Mota and Depraz [15] found that male Serin *Serinus serinus* vocal behavior did not change in response to playback. Mennill et al. [14] showed that chickadee responses depended on the details of the stimulus and status of the male; high-ranking males lost paternity from aggressive playback (matched pitch and overlapped songs), but not submissive playback, and playback did not change paternity in low-ranking males. Wingfield et al. [12] reviewed the literature and found that hormonal responses to playback tended to depend on breeding strategy, with monogamous species showing the strongest responses and polygynous species (males uninvolved in parental care) showing the weakest responses. For example, male polygynous Japanese Bush-warblers *Cettia diphone* showed no increase in plasma corticosterone in response to playback [16]. Additionally, visual as well as auditory stimuli may be required to elicit hormonal changes [17]. In a potentially positive case, Mota and Depraz [15] found that female Serins spend 35% more time nest building in response to playback of stranger male song, which could potentially lead to faster nest completion and higher fitness, given that earlier hatched young may have higher survival (e.g. [18]). In a final case, Ward and Schlossberg [19] found that playing song of the imperiled Black-capped *Vireo Vireo atricapilla* for 6.5 hours daily over 3.5 months can attract vireos to vacant territories. The new territories resulted in successful nesting and appeared to lead to population increases. Interestingly, the vireos appeared to regard the continued playback as small territories and habituated to the playback as they would normal neighbors [19].

The potential effects of playback on birds have been reviewed by several popular articles (e.g. [7,10]), and hotly debated on ornithology and birdwatching forums such as Neotropical Ornithology (http://www.museum.lsu.edu/~Remsen/NEOORNintro.html) and Birding Australia (http://birding-aus.org/), but no peer-reviewed studies have examined the effects of birdwatchers’ playback on birds. Despite this lack of information, several organizations have assumed playback is harmful. For example, playback was characterized as a substantial threat to species of conservation concern in the United States, including Rose-throated Becard *Pachyramphus aglaiae* [20,21], and Kirtland’s Warbler *Dendroica kirtlandii* [22]. The American Birding Association’s “Code of ethics” also calls for limited use of playback [23]. Furthermore, Sen [10] found that many regional and species-specific playback restrictions have been enacted. Two of the many restrictions detailed by Sen [10] are the United Kingdom’s Wildlife and Countryside Act’s rule against playback of all birds listed in schedule 1 [24], and Melbourne Water’s ban on playback for any species of crake at the Western Treatment Plant, Australia [25]. Until the effects of playback are objectively evaluated, restrictions on birdwatchers’ behavior may be poorly justified. Furthermore, if negative impacts are identified, then regulations should be guided by quantified costs and benefits of birdwatching activity.

 In this paper we first evaluate the effects of simulated birdwatchers’ playback on vocal behavior of Plain-tailed Wrens *Thryothorus euophrys* and Rufous Antpittas *Grallaria rufula*. Secondly, we test for changes in response over time with repeated playback trials in Plain-tailed Wrens. Considering the results of previous playback studies, we predicted that both wrens and antpittas would change their vocal behavior in response to playback, and that strength of wren response would decrease over time in the repeated trials due to habituation.

## Materials and Methods

### Study site

From 15 September to 15 November 2006, we (fieldwork done by J.B.C.H.) studied the effects of (1) single bouts of playback on vocal behavior of 24 groups of Plain-tailed Wrens and 12 groups of Rufous Antpittas, and (2) repeated playback over 20 days in five wren groups. We worked in Fundación Jocotoco’s Tapichalaca Biological Reserve (4°29’S, 79°07’W; 2,400–2,600 m.a.s.l.) in Zamora-Chinchipe province, southern Ecuador. The upper subtropical forest in the area has an average canopy height of approximately 10 m, with 20 m tall emergent crowns, and receives c. 4,000 mm of rainfall annually [26].

We began by spending two weeks covering much of the reserve to find sites where Plain-tailed Wrens and Rufous Antpittas consistently vocalized. If an individual was heard vocalizing on at least two days from the same site, it was considered to comprise a resident group. The average distance to the nearest neighboring group was 195 m ± 89 SD for wrens and 389 m ± 159 SD for antpittas. The study period coincided with a peak of breeding activity for many species in the reserve, including wrens and antpittas [27,28].

### Single bout experiments

In this study our goal was to mimic playback by birdwatchers, where a short bout of song is played to elicit a territorial response. Birdwatchers’ playback can involve self or stranger song played anywhere from a few seconds to a few hours (J.B.C.H. pers. obs.). In self song playback, birdwatchers record a vocalizing bird and play the vocalization. On the other hand, stranger song playback (which we used in this study) usually involves birdwatchers playing vocalizations of the same species recorded at another site (from a bird song CD or online source). Technically, “playback” should refer to self song playback alone, but here we use the word to refer to self and stranger song broadcast, as is done in the birdwatching community. Our five minute treatments might be characterized by many birdwatchers as the “judicious use of playback” of the kind that is thought to have minor effects on bird behavior [7].

For the single bout experiments we monitored the vocal behavior of 24 groups of wrens and 12 groups of antpittas for an hour after playing five minutes of song (“playback treatment”) and after a five minute control treatment of background noise. Background noise broadcast was used to control for the effects of disturbance by the observer and noise from the speaker. Trials were done from 5:50–11:00, except during heavy rain or wind (greater than 15 km/hr) [29]. Recordings were made with a Sennheiser ME 66 shotgun microphone and an M-Audio Microtrack digital recorder. Stimuli were broadcast with a small portable speaker and standardized using a sound pressure meter at approximately 60 decibels. J.B.C.H. sat 50 cm from the speaker during the experiments to simulate a birdwatcher.

Treatment (playback) stimuli were randomly selected from song recordings of five groups of each study species that had territories in distant parts of the reserve and were not included in the study. Thus, all stimuli were stranger song and none were neighbors of study groups. Control stimuli were randomly selected from five recordings of background noise in the reserve. These recordings were made in the afternoon and included only insect sounds and wind noise, with no bird vocalizations. We provide sonograms and samples of treatment and background stimuli, and summary measurements of treatment stimuli in the Supporting Information (Tables S1 and Figures S1–S3 in File S1; supporting sound files S1–S3). We measured low, center, high, peak, and delta frequency (Hz) as well as delta time (s) in Raven Pro 1.5 Build 11 (2012, Bioacoustics Research Program, Cornell Laboratory of Ornithology), using spectrograms with a Hann window, and a 3 dB filter with the bandwidth set at 90.6 Hz. All measurements were taken from fundamental frequencies (not from harmonics) of individual phrases (n = 30 per species, 6 phrases for 5 different stimuli). 

We measured playback response by comparing the number of vocalizations produced and the number of repetitions of each vocalization after each playback trial compared to the same variables after control trials. All data were recorded in real time in the field in a notebook. We defined vocalizations as cohesive series of notes separated by a period of silence. Repetitions were the number of notes in each vocalization. Definition of these variables depended on the species and vocalization type (see below). Increasing the number of vocalizations or the number of repetitions per vocalization are positively correlated with the costs of singing [30,31].

Rufous Antpittas probably form monogamous groups comprised of one male and one female [32]. We never saw more than two individuals together. The most commonly heard vocalization of *Grallaria* r. *rufula* at our study site was the *short song* (referred to as “alternative song” in Krabbe and Schulenberg [32]) that consists of a loud note followed by 4–5 lower pitched, descending, accelerating notes (see recording XC17548 by A. Spencer at http://www.xeno-canto.org/). Rufous Antpittas also produced *long songs* (longer versions of short songs; J. King recording XC101056) and ringing *trills* (c. 2 sec long, c. 20 notes; N. Athanas recording XC32383) [32]. The functions of these different vocalizations types are apparently unstudied. Recordings of *short songs* were used as stimuli in antpitta playback experiments because they are commonly heard and likely are used in territorial interactions (J.B.C.H. pers. obs.). The number of *short songs*, *long songs*, *trills*, and *total vocalizations*, as well as the number of repetitions per vocalization for each type, were the response variables for the antpitta experiments.

Plain-tailed Wrens form groups of 2–7 individuals comprised of a pair and its offspring [33]. Wren groups sing exceptionally complex songs with four-part, synchronized, chorusing *duets* (ref [33].; recording XC4198 by W. Halfwerk). At our study site, duets were the most commonly heard vocalization from *Thryothorus euophrys longipes* (subspecies taxonomy follows Ridgely and Greenfield [34]). Duets in this species are hypothesized to be used for territorial defense and/or to synchronize reproductive efforts in the group [33]. Plain-tailed Wrens also produced *double contact calls* (paired “choo-chip” vocalizations; W. Halfwerk recording XC4199), *melody songs* (songs similar to duet phrases but produced by single birds at lower volume and well separated by pauses; F. Lambert recording XC38858), and fast, harsh *chatters* (L. Ordóñez-Delgado recording XC78746) [35]. Recordings of *duets* were used as stimuli in wren playback experiments; as in the antpitta experiments, we selected a commonly heard vocalization type that likely serves to maintain territorial boundaries. The response variables for Plain-tailed Wrens were the number of *duets*, *double contact calls*, *melody songs*, *chatters*, and *total vocalizations*, and repetitions per vocalization for each type. We also grouped all *non-duet vocalizations* as a response variable because these vocalizations were quieter, produced by single birds, and likely to differ in function from duets. 

 We used Gaussian general linear models in a maximum likelihood framework to test for playback-induced changes in vocal behavior. For each response variable, we used Akaike’s Information Criterion corrected for small sample sizes to compare support for group models (playback vs. control) to null models [36,37]. For all analyses we examined diagnostic plots that show the relationship between the fitted values and residuals, the quantiles in the data against theoretical normal quantiles, and the relationship between leverage and standardized residuals for all fitted models, and found that all assumptions of the Gaussian error struture were met [38]. All statistical modeling was done in R v.2.14.1 [39]. 

### Repeated playback experiments

In the second part of the study, we monitored the effects of daily playback on five groups of Plain-tailed Wrens. Study groups were randomly selected from groups studied in the single bout experiments. Two groups had two individuals; the other three groups had three individuals. As in the single bout experiments, we played song stimuli for five minutes and recorded bird responses for an hour. Experiments were done from 11:10–15:00 over 20 consecutive days, and each group received playback at the same time daily (± 20 minutes). Each group received different stimuli (see ‘single bout experiments’), but the same stimuli each day. The study groups for the repeated experiments received their single bout treatments on 13–17 October, and the repeated experiments began on 19 October. Groups were located far enough apart (320–840 m) that playback at one group was inaudible to others.

We used three metrics to gauge responses to repeated playback: closeness of approach to the speaker, latency of vocal response (time to first vocalization), and latency of visual response (time to first sighting of a responding bird). On the first day, we used a tape measure to quantify the distance from the speaker to where the bird approached. Subsequently we used flagging to label radii from the speaker at 2, 5, 8, 10, 20, 30, 40, and 50 m, in order to facilitate distance estimates. We recorded responses for 60 minutes after the start of playback; if none of the study birds were seen or heard, the maximum distance (50 m) or time (3600 sec) were recorded, respectively. There was no control in the repeated playback experiments (we only recorded closeness of approach or latency of response after playback). Again, all data were recorded in a field notebook.

We used Gaussian mixed-effect models with log-transformed response variables to compare **playback response ~ time** to **playback response ~ null** models. Non-responses in the repeated playback experiments lead to violated assumptions for ordinary Gaussian general linear models (strong patterns in residuals of fitted models). We corrected this by natural log transforming the response variables [38]. Mixed-effect modeling is an appropriate method to account for correlations in repeated measurement datasets, such as in the current study where we repeatedly played songs to the same groups [40]. Following Zuur et al. [40], we checked the support for using mixed-effect models by comparing global models fit with generalized least squares regression, random intercept (wren group as the random effect), and random slope (time | group) in the nlme package [41]. We used AIC calculated with restricted likelihood to compare the models [40], and also checked the support for a random intercept with restricted likelihood ratio tests in the RLRsim package [42]. There was strong support for mixed-effect models over generalized least squares regression, and random intercept was top-ranked for all three response variables (Δ AIC of random intercept model ranged from 3.6–26.8 compared to second highest ranked model; restricted likelihood ratio test *P* < 3x10^-16^ for random intercept). For the final analysis we compared **playback response ~ time + (1 | group**), **playback response ~ 1 + (1 | group**), **and playback response ~ 1 + (1 | null**), where “null” was a column of 1s, to evaluate the relative impacts of the fixed and random effects in the lme4 package [43]. Model diagnostics showed the data generally met the necessary assumptions for Gaussian models. Nonetheless, trends in the residuals and minor departure from normality for latency of visual response are reasons for caution in interpretation.

## Results

**Figures 1:**
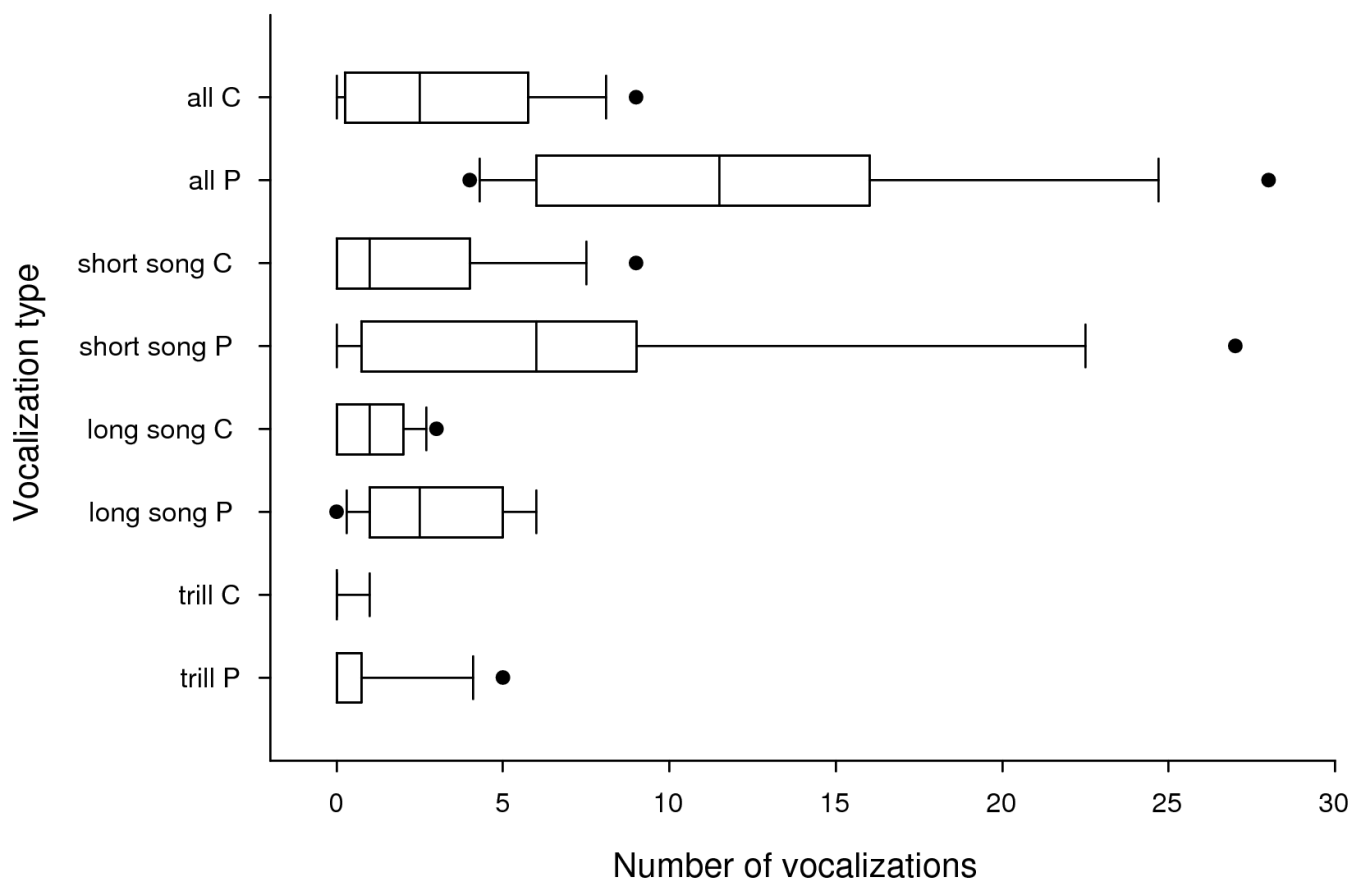
Boxplots of vocalizations produced by 12 groups of Rufous Antpittas *Grallaria rufula* in an hour after playback (P) and control (C) treatments. Boxes show the 25^th^ and 75^th^ percentiles, the center line shows the median, whiskers show the 10^th^ and 90^th^ percentiles, and points show outliers. Antpittas produced more short songs, long songs, and total vocalizations, but not trills, after playback.

**Figures 2:**
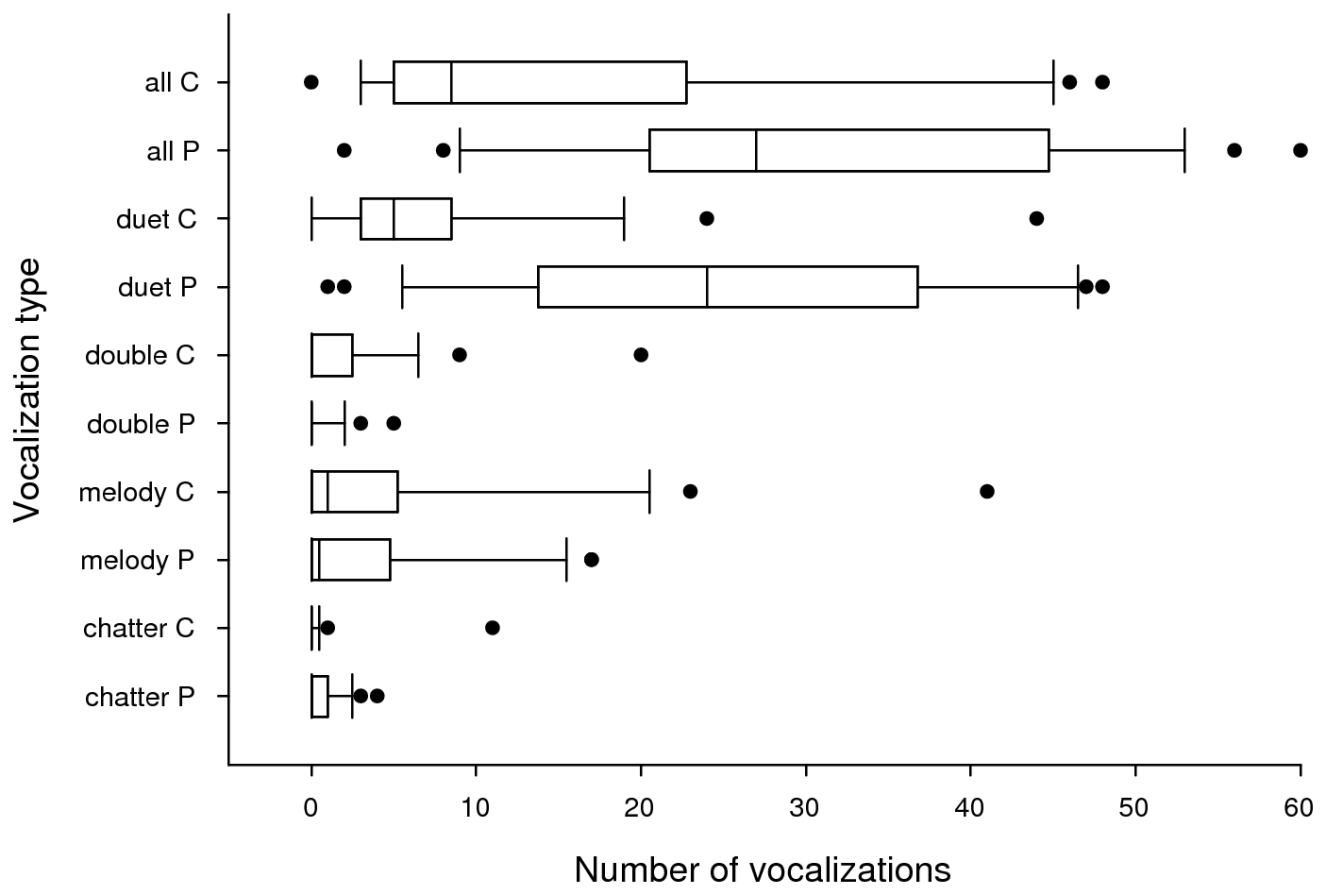
Boxplots of vocalizations produced by 24 groups of Plain-tailed Wrens *Thryothorus euophrys* in an hour after playback (P) and control (C) treatments. Boxes show the 25^th^ and 75^th^ percentiles, the center line shows the median, whiskers show the 10^th^ and 90^th^ percentiles, and points show outliers. Wrens produced more duets and total vocalizations, but not other vocalization types after playback.

Both Rufous Antpittas and Plain-tailed Wrens changed their vocal behavior in response to a single bout of simulated birdwatchers’ playback. Rufous Antpittas produced more short songs, long songs, and total vocalizations, but not more trills, after playback treatments compared to controls (Figures 1, Tables 1). Evidence for a difference in the number of vocalizations between treatments was strong for total vocalizations (*w*AIC_*c*_ for the group model of 0.996) and long songs (wAIC_*c*_ = 0.939), but rather weak for short songs (wAIC_*c*_ = 0.703). Antpittas also produced more repetitions per vocalization after playback than after controls for the same three vocalization types (*w*AIC_*c*_ 0.708–0.999; Table S2 in File S1). Plain-tailed Wrens produced more duets and total vocalizations after playback, but there was no difference for other vocalization types (Figures 2, Tables 2). Evidence was strong for both duets (wAIC_*c*_ = 1.0) and total vocalizations (wAIC_*c*_ = 0.984). The large difference in the number of duets produced after playback likely had a substantial impact on the total vocalizations result. Wrens produced more repetitions per vocalization only in duets (wAIC_*c*_ = 1.0; Table S3 in File S1). 

**Tables 1:** Evidence for playback-induced changes in the number of short songs, long songs, trills, and all vocalizations in Rufous Antpitta *Grallaria rufula* produced in an hour period.

Model	Δ AIC_*c*_	*w_i_*	*k*	% DE
*all vocalizations*				
group	0^a^	0.996	3	43.7
null	11.1	0.004	2	0
*short songs*				
group	0	0.703	3	16.6
null	1.7	0.297	2	0
*long songs*				
group	0	0.939	3	28.6
null	5.5	0.061	2	0
*trills*				
null	0	0.657	2	0
group	1.3	0.343	3	5.4

All song types except trills increased after playback (control vs. playback group effect ranked above null). Δ AIC_*c*_ shows the difference between the model AIC_*c*_ (Akaike’s Information Criterion corrected for small sample sizes) and the minimum AIC_*c*_ in the set of models; AIC_*c*_ weights (*w*_*i*_) show the relative likelihood of model *i*; *k* indicates the number of parameters; % DE is percent deviance explained by the model.

^a^ Lowest AIC_*c*_ = 152.7 (all vocalizations), 156.5 (short songs), 99.2 (long songs), and 76.2 (trills).

**Tables 2:** Evidence for playback-induced changes in the number of three individual vocalization types, non-duet vocalizations, and all vocalizations in Plain-tailed Wren *Thryothorus euophrys* produced in an hour period.

Model	Δ AIC_*c*_	*w_i_*	*k*	% DE
*all vocalizations*				
group	0^a^	0.984	3	19.8
null	8.3	0.016	2	0
*non-duet vocalizations*				
null	0	0.673	2	0
group	1.4	0.327	3	1.7
*duets*				
group	0	1	3	35.5
null	18.8	0	2	0
*double contact calls*				
null	0	0.506	2	0
group	0.05	0.494	3	4.5
*chatters*				
null	0	0.747	2	0
group	2.2	0.253	3	0.23
*melody songs*				
null	0	0.721	2	0
group	1.9	0.279	3	0.8

Duets and all vocalizations increased after playback (control vs. playback group effect ranked above null). Δ AIC_*c*_ shows the difference between the model AIC_*c*_ (Akaike’s Information Criterion corrected for small sample sizes) and the minimum AIC_*c*_ in the set of models; AIC_*c*_ weights (*w*_*i*_) show the relative likelihood of model *i*; *k* indicates the number of parameters; % DE is percent deviance explained by the model.

^a^ Lowest AIC_*c*_ = 401.8 (all vocalizations), 264.7 (non-duet vocalizations), 377.6 (duets), 253.0 (double contact calls), 193.1 (chatters), and 339.4 (melody songs).

**Figures 3:**
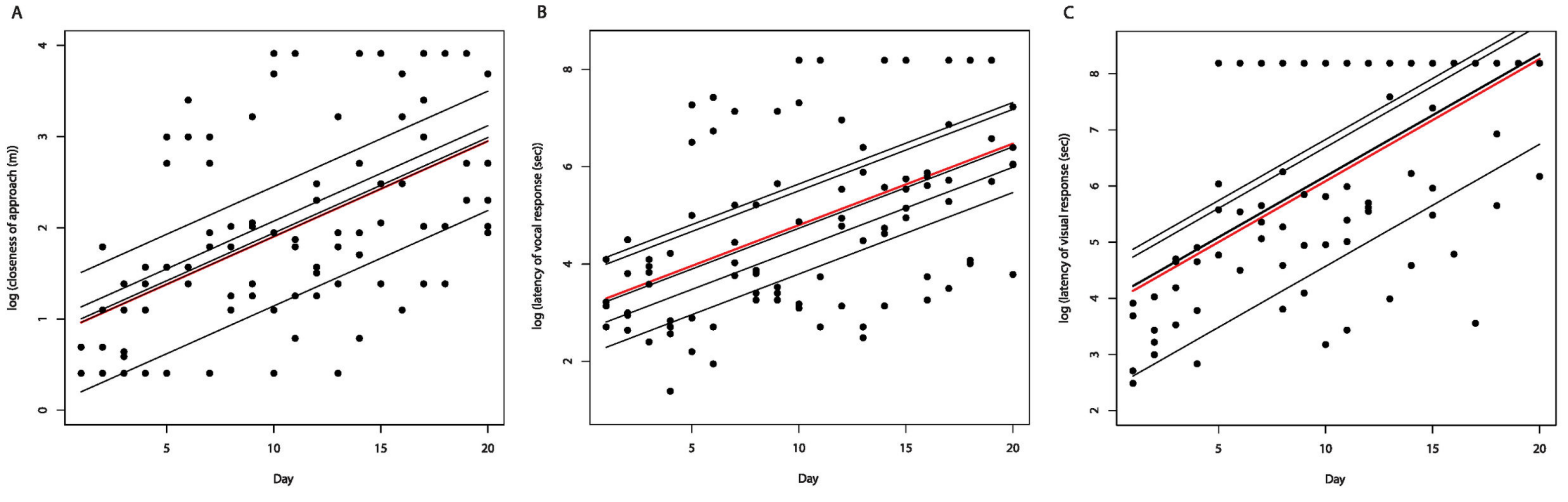
In repeated playback experiments, Plain-tailed Wren *Thryothorus euophrys* groups showed weaker responses over time measured by (a) closeness of approach to the speaker, (b) latency of vocal response, and (c) latency of visual response. In each figure the red line shows the population trend line (all wren groups combined) and the other lines show fitted trend lines for each group from Gaussian mixed-effect models with random intercepts. Data points with maximum values on the y-axis indicate no response (i.e. latency of response of 3,600 seconds or closeness of approach of 50 m).

 In repeated playback experiments, the five Plain-tailed Wren groups showed reduced responses to playback over time, indicating habituation (Figures 3, Tables 3). Wrens responded strongly at first, but responses had already begun to decline by day five, and after approximately day 12, wrens showed little or no response. In Figures 3, data points with maximum values on the y-axis indicate no response (i.e. latency of response of 3,600 seconds or closeness of approach of 50 m). Evidence of declining responses over time was strong for all three response variables (wAIC_*c*_ = 1.0 for time model). Fitted trend lines for each wren group were widely spaced in all repeated playback response variables, which agrees with the strong support for random intercept models we observed (Figures 3).

**Tables 3:** Evidence for habituation in five Plain-tailed Wren *Thryothorus euophrys* groups that experienced playback once a day for 20 days.

Model	ΔAIC_*c*_	*w_i_*	*k*	% DE
*closeness of approach*				
time + (1 | group)	0	1.0	4	17.2
null + (1 | group)	38.4	0.0	3	3.6
null + (1 | null)	49.1	0.0	3	0
*latency of vocal response*				
time + (1 | group)	0	1.0	4	10.4
null + (1 | group)	30.3	0.0	3	2.4
null + (1 | null)	40.0	0.0	3	0
*latency of visual response*				
time + (1 | group)	0	1.0	4	18.8
null + (1 | group)	64.0	0.0	3	2.8
null + (1 | null)	75.5	0.0	3	0

Birds waited longer to respond and did not approach the speaker as closely over time. The random effect “null” is a column of 1s.

 Interestingly, one of the wren groups built a nest approximately 10 m from the speaker site during the study. We observed the birds carrying nesting material during the song treatment on days 13 and 14, apparently unaffected by the playback.

## Discussion

Rufous Antpittas produced more songs, but not trills, and Plain tailed-Wrens produced more duets, but not other vocalization types, after simulated birdwatchers’ playback compared to controls. In addition, antpittas and wrens produced more repetitions per vocalization after playback for the same vocalization types. These results suggest that birdwatchers’ playback affects vocal behavior of these two tropical passerines. Our findings agree with previous studies that indicated playback in a non-birdwatching context can affect avian vocal behavior (e.g. [14]). 

Playback affected the delivery rate of duets but had no effect on contact calls or other non-duet vocalizations in wrens. It is possible that these differences were caused by the function of these different vocalizations. Contact calls serve as intragroup signals and might therefore not be expected to increase during the territorial incursion that playback simulates. 

The changes in vocal behavior and habituation we observed suggest that scientists may need to consider the effects of birdwatchers’ playback when censusing populations or designing behavioral experiments. It may be important to study naïve bird populations that do not experience frequent birdwatching in order to work with baseline conditions. Accordingly, Lima and Roper [44] found that naïve passerines in Brazil responded more eagerly than birds exposed to frequent playback over approximately three weeks a month earlier. 

The repeated playback experiments suggest that Plain-tailed Wrens may habituate to repeated short bouts of birdwatchers’ playback after just 12 days of playback. This finding suggests that repeated playback may not have stronger effects on wren vocal behavior than single bout playback. It is possible that repeated short bouts of birdwatchers’ playback could lead to birds treating playback as normal neighbors, as was apparently the case in Ward and Schlossberg’s [19] long-term experiments. The wren nest building that we observed near the playback speaker supports this possibility. Habituation could explain why particular bird pairs that are repeatedly targeted by birdwatchers with playback stop responding and seem to “disappear” [45]. Considering the above, irregular playback could potentially have a greater impact on bird behavior if individuals do not encounter playback often enough to habituate, and respond strongly in each instance of playback. On the other hand, if habituated birds show less pronounced responses, they might be less effective at defending their territories from true rivals [46]. These alternative hypotheses require further investigation.

Our findings are from a limited sample of 12 groups of antpittas and 24 groups of wrens. Furthermore, playback impacts may vary depending on taxonomic group, song complexity, social behavior, and time of year (e.g. [12]), so additional studies in other taxa are needed to establish the generality of our findings. Although our data show that bird behavior changes in response to playback, we did not measure the effects of playback on components of fitness such as survival or reproductive success. 

Our results indicate that birdwatchers’ playback affects the vocal behavior of two species of Neotropical songbirds. This result suggests that playback could negatively affect species if they become stressed, expend energy, or take time away from other activities to respond to playback. By contrast, the habituation results we present suggest that frequent birdwatchers’ playback may have minimal impacts on wren behavior.

## Supporting Information

File S1 Table S1, Summary measurements of stimuli used for playback experiments. Table S2, Evidence for playback-induced changes in the number repetitions per vocalisation in Rufous Antpitta *Grallaria rufula*. Table S3, Evidence for playback-induced changes in the number repetitions per vocalisation in Plain-tailed Wren *Thryothorus euophrys*. Figure S1, Sonograms of samples of five stimuli used for Rufous Antpitta *Grallaria rufula* playback treatments. Figure S2, Sonograms of samples of five stimuli used for Plain-tailed Wren *Thryothorus euophrys* playback treatments. Figure S3, Sonograms of samples of five background noise recordings broadcast in single bout experiments.
(DOCX)

Sound File S1 **Rufous Antpitta *Grallaria rufula* audio stimulus sample.**
(WAV)

Sound File S2 **Plain-tailed Wren *Thryothorus euophrys* audio stimulus sample.**
(WAV)

Sound File S3 **Background noise sample.**
(WAV)
