# Supplementary material for: Simulated Birdwatchers’ Playback Affects the Behavior of Two Tropical Birds
Source: PLoS One. 2013 Oct 11;8(10):e77902. doi: 10.1371/journal.pone.0077902 (PMC3797570; doi:10.1371/journal.pone.0077902)
Supplement: File S1 — Table S1, Summary measurements of stimuli used for playback experiments. Table S2, Evidence for playback-induced changes in the number repetitions per vocalisation in Rufous Antpitta Grallaria rufula. Table S3, Evidence for playback-induced changes in the number repetitions per vocalisation in Plain-tailed Wren Thryothorus euophrys. Figure S1, Sonograms of samples of five stimuli used for Rufous Antpitta Grallaria rufula playback treatments. Figure S2, Sonograms of samples of five stimuli used for Plain-tailed Wren Thryothorus euophrys playback treatments. Figure S3, Sonograms of samples of five background noise recordings broadcast in single bout experiments. (DOCX) [file pone.0077902.s001.docx]

**Supporting Information**

Table S1. Summary measurements of stimuli used for playback experiments (mean ± SD). Measurements were taken from fundamental frequencies of individual phrases (n = 30 per species, 6 phrases for 5 different stimuli).

| Stimulus | Low frequency (Hz) | High frequency (Hz) | Peak frequency (Hz) | Delta Time (s) | Center frequency (Hz) | Delta frequency (Hz) |
| --- | --- | --- | --- | --- | --- | --- |
| Wren 1 | 920.4 ± 64.6 | 3696.9 ± 179.9 | 2153.4 ± 344.5 | 1.3 ± 0.1 | 2124.6 ± 215.6 | 2776.6 ± 118 |
| Wren 2 | 983.1 ± 163.4 | 3770.7 ± 241.6 | 2153.3 ± 719.1 | 1.3 ± 0.5 | 1880.6 ± 285.3 | 2787.7 ± 296.5 |
| Wren 3 | 621.3 ± 37.9 | 3664.3 ± 184.3 | 2062.5 ± 233.5 | 1.3 ± 0.2 | 2062.5 ± 51.3 | 3043 ± 150.3 |
| Wren 4 | 630.8 ± 66.6 | 3466.2 ± 29.1 | 1945.3 ± 150.5 | 1.4 ± 0.3 | 2078.1 ± 24.2 | 2835.4 ± 87.4 |
| Wren 5 | 573.2 ± 32.6 | 3584 ± 38.3 | 1859.4 ± 105.5 | 1.4 ± 0.2 | 2070.3 ± 80.7 | 3010.7 ± 46.8 |
| Antpitta 1 | 1020.7 ± 51.5 | 3067.2 ± 145.3 | 2483.5 ± 58.8 | 0.6 ± 0 | 2418.9 ± 32.4 | 2046.4 ± 151.1 |
| Antpitta 2 | 1148 ± 63.5 | 3018.8 ± 77.4 | 2275.4 ± 32.4 | 0.6 ± 0 | 2239.5 ± 27.2 | 1870.7 ± 101.3 |
| Antpitta 3 | 1946 ± 11.8 | 2503.1 ± 27.9 | 2153.3 ± 0 | 0.7 ± 0 | 2196.4 ± 0 | 557.1 ± 28.9 |
| Antpitta 4 | 1824.1 ± 11.4 | 3192.4 ± 239.8 | 2325.6 ± 27.3 | 0.6 ± 0 | 2282.5 ± 0 | 1368.3 ± 235.1 |
| Antpitta 5 | 1934.2 ± 35.5 | 2690.5 ± 87.4 | 2347.2 ± 23.6 | 0.6 ± 0 | 2325.6 ± 0 | 756.3 ± 101.3 |

Table S2. Evidence for playback-induced changes in the number repetitions per vocalisation for short songs, long songs, trills, and all vocalisations in Rufous Antpitta *Grallaria rufula* produced in an hour period. In antpittas, repetitions per vocalisation were higher for all vocalisation types except trills after playback (control vs. playback group effect ranked above null). Δ AIC*_c_* shows the difference between the model AIC*_c_* (Akaike’s Information Criterion corrected for small sample sizes) and the minimum AIC*_c_* in the set of models; AIC*_c_* weights (*w_i_*) show the relative likelihood of model *i*; *k* indicates the number of parameters; % DE is percent deviance explained by the model.

| Model | Δ AIC*_c_* | *w_i_* | *k* | % DE |
| --- | --- | --- | --- | --- |
| *all vocalisations* |  |  |  |  |
| group | 0^a^ | 0.999 | 3 | 49.9 |
| null | 14.0 | 0.001 | 2 | 0 |
| *short songs* |  |  |  |  |
| group | 0 | 0.980 | 3 | 35.3 |
| null | 7.8 | 0.020 | 2 | 0 |
| *long songs* |  |  |  |  |
| group | 0 | 0.708 | 3 | 16.7 |
| null | 1.8 | 0.292 | 2 | 0 |
| *trills* |  |  |  |  |
| null | 0 | 0.710 | 2 | 0 |
| group | 1.8 | 0.290 | 3 | 3.4 |

^a^Lowest AIC*_c_* = 112.8 (all vocalisations), 144.5 (short songs), 44.5 (long songs), and 53.9 (trills).

Table S3. Evidence for playback-induced changes in the number of repetitions per vocalisation for three individual vocalisation types, non-duet vocalisations, and all vocalisations in Plain-tailed Wren *Thryothorus euophrys* produced in an hour period. Only duets had higher repetitions per vocalisation after playback (control vs. playback group effect ranked above null). Δ AIC*_c_* shows the difference between the model AIC*_c_* (Akaike’s Information Criterion corrected for small sample sizes) and the minimum AIC*_c_* in the set of models; AIC*_c_* weights (*w_i_*) show the relative likelihood of model *i*; *k* indicates the number of parameters; % DE is percent deviance explained by the model.

| Model | ΔAIC*_c_* | *w_i_* | *k* | % DE |
| --- | --- | --- | --- | --- |
| *all vocalisations* |  |  |  |  |
| null | 0^a^ | 0.724 | 2 | 0 |
| group | 1.9 | 0.276 | 3 | 0.7 |
| *non-duet vocalisations* | |  |  |  |
| null | 0 | 0.743 | 2 | 0 |
| group | 2.1 | 0.257 | 3 | 0.3 |
| *duets* |  |  |  |  |
| group | 0 | 1 | 3 | 7.4 |
| null | 1.4 | 0 | 2 | 0 |
| *double contact calls* | |  |  |  |
| null | 0 | 0.678 | 2 | 0 |
| group | 1.5 | 0.322 | 3 | 1.6 |
| *chatters* |  |  |  |  |
| null | 0 | 0.669 | 2 | 0 |
| group | 1.4 | 0.331 | 3 | 1.8 |
| *melody songs* |  |  |  |  |
| null | 0 | 0.723 | 2 | 0 |
| group | 1.9 | 0.277 | 3 | 0.7 |

^a^Lowest AIC*_c_* = 270.3 (all vocalisations), 337.1 (non-duet vocalisations), 269.5 (duets), 337.2 (double contact calls), 313.1 (chatters), and 210.1 (melody songs).


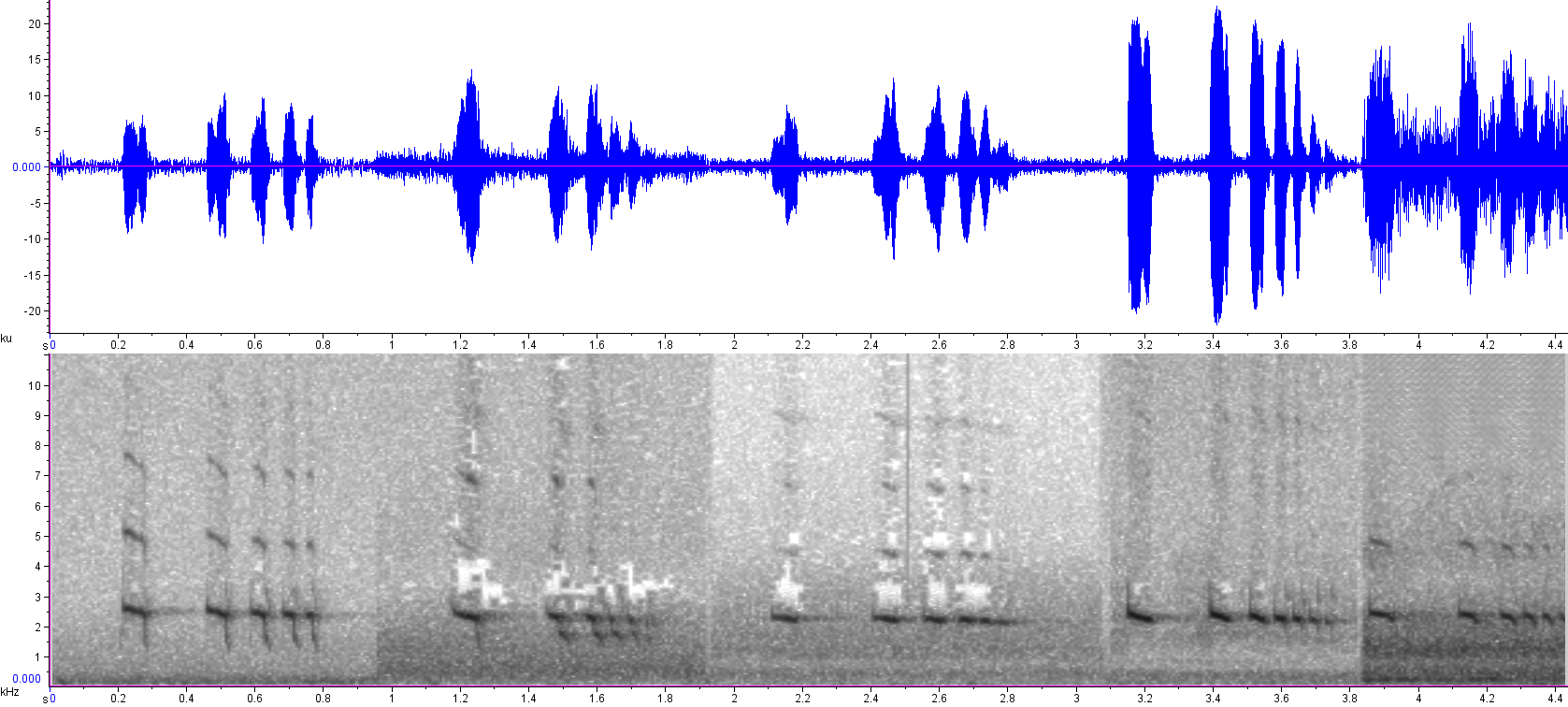


Fig. S1. Sonograms of samples of five stimuli used for Rufous Antpitta *Grallaria rufula* playback treatments. Spectrograms were made in Raven Pro using a Hann window, with 75% overlap on the time grid and 25.1 Hz on the frequency grid.


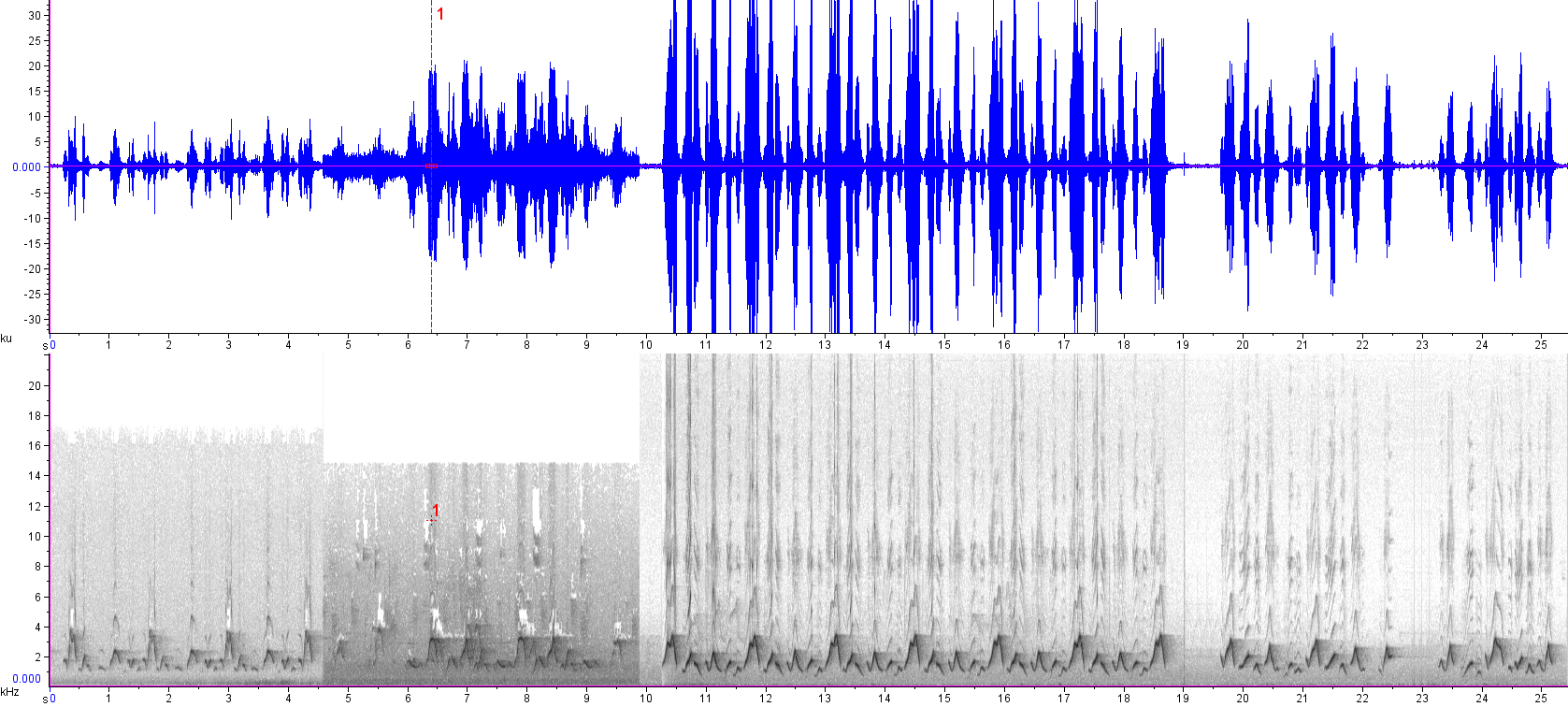


Fig S2. Sonograms of samples of five stimuli used for Plain-tailed Wren *Thryothorus euophrys* playback treatments. Spectrograms were made in Raven Pro using a Hann window, with 75% overlap on the time grid and 25.1 Hz on the frequency grid.


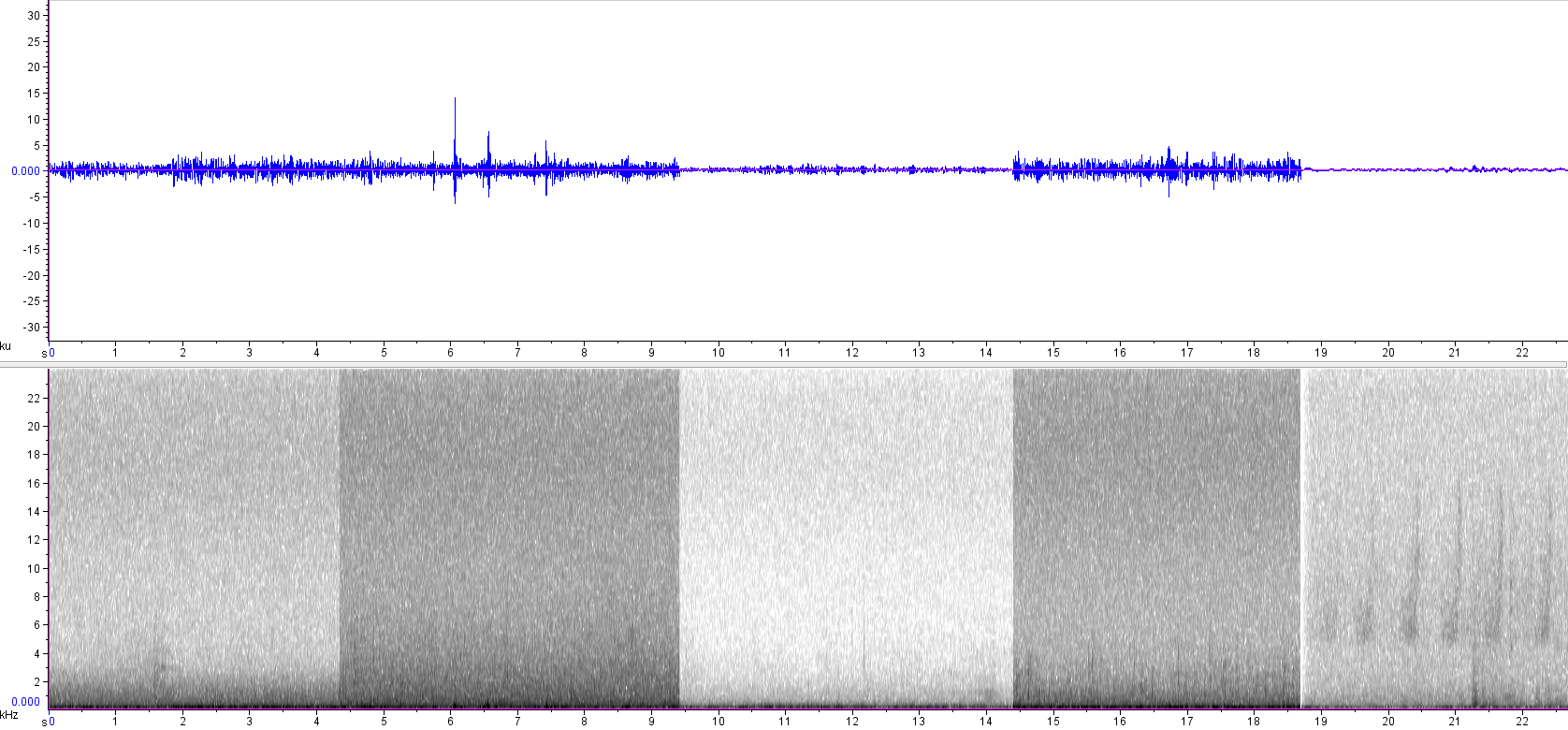


Fig. S3. Sonograms of samples of five background noise recordings that were broadcast in the single bout experiments. Spectrograms were made in Raven Pro.
